# Biological function of eosinophil extracellular traps in patients with severe eosinophilic asthma

**DOI:** 10.1038/s12276-018-0136-8

**Published:** 2018-08-16

**Authors:** Youngwoo Choi, Duy Le Pham, Dong-Hyun Lee, So-Hee Lee, Seung-Hyun Kim, Hae-Sim Park

**Affiliations:** 1 Department of Allergy and Clinical Immunology, Ajou University School of Medicine, Suwon, South Korea; 2 Faculty of Medicine, University of Medicine and Pharmacy, Ho Chi Minh, Vietnam; 3 Clinical Trial Center, Ajou University Medical Center, Suwon, South Korea

## Abstract

Eosinophil extracellular traps (EETs), a complex of DNA fibers and cytotoxic granule proteins, are implicated in the development of asthma; however, the pathophysiological function of EETs in immune responses has not been fully determined. The present study investigated the characteristics of EETs from patients with non-severe asthma (NSA, *n* = 20) and severe eosinophilic asthma (SEA, *n* = 20) and evaluated EET function. The percentage of EET-forming peripheral blood eosinophils stimulated with IL-5 and LPS was significantly higher in patients with SEA than in those with NSA *(P* = 0.009). This percentage negatively correlated with baseline FEV_1_ (*r* = −0.350, *P* = 0.027) and positively correlated with serum eosinophil-derived neurotoxin levels in asthmatic subjects (*r* = 0.437, *P* = 0.018). In addition, EET formation was markedly associated with reactive oxygen species production (*r* = 0.750, *P* *<* 0.001). These EETs exhibited an autocrine function to induce eosinophil degranulation, which led to granule protein production. Airway epithelial cells stimulated with EETs exhibited increased epithelial detachment and permeability and pro-inflammatory cytokine release. However, EETs were not significantly associated with mast cell activation. The present study suggests that peripheral blood eosinophils from patients with SEA may be more activated to produce EETs than those from patients with NSA, which further induces inflammation in asthmatic airways. Therefore, regulation of EET formation and function may be a novel therapeutic approach for asthma management.

## Asthma: Traps to target for treatment

Controlling the formation of mesh-like structures of DNA and protein which induce airway damage in asthma could offer new therapeutic opportunities, especially for patients who do not respond well to conventional treatments. The structures are produced by activated cells as part of the immune response in asthma. They are known as eosinophil extracellular traps (EETs) since they are most prominently released by the activity of white blood cells called eosinophils. Hae-Sim Park and colleagues at Ajou University in South Korea studied the involvement of EETs in patients with severe asthma. Their results suggest eosinophils are more activated to create damaging EETs in patients with severe asthma than in those with less severe forms of the disease. Searching for ways to control EET formation could be a fruitful approach to helping patients with severe eosinophilic asthma.

## Introduction

Asthma is a complicated airway disease with bronchial hyperresponsiveness^1^. The disease exists worldwide in a broad spectrum of phenotypes, and 300 million people are affected. However, ~10% of asthmatic patients with severe symptoms are poorly controlled with conventional treatment^2,3^. These patients experience frequent asthma exacerbations with morbidity/mortality and consume more healthcare resources, which results in hospital admissions and work loss^4^. Several studies investigated the pathophysiology of severe eosinophilic asthma (SEA), but a distinct immunological pathway was not fully determined because asthma is highly heterogeneous.

Eosinophilic inflammation is a key feature of asthma severity because it generates lung dysfunction^5,6^. Eosinophils play an important role in innate immunity during parasitic and helminthic infections^7^. However, whether these cells are beneficial in asthma is not certain because substantial evidence has demonstrated a role for eosinophils in tissue damage and asthma symptoms^8^. Eosinophilia generates airway inflammation and remodeling, which are closely associated with asthma exacerbation in SEA^9–11^. Eosinophil-derived granule proteins involved in immune responses, such as major basic protein, eosinophil cationic protein (ECP) and eosinophil-derived neurotoxin (EDN), were extensively studied^12–14^. High levels of granule proteins are indicative of eosinophil activation and degranulation, which is associated with SEA.

Eosinophil extracellular traps (EETs) are a complex meshwork of DNA fibers and granule proteins that play a role in innate immunity to infectious disease and induce tissue damage in asthmatic airways^15–18^. Eosinophils release EETs in a NADPH oxidase-dependent manner associated with reactive oxygen species (ROS) production^19–21^. Neutrophils also produce extracellular traps via rapid cell death^22,23^. The pathogenic or harmful effect of an excess of neutrophil extracellular traps (NETs) was investigated^24,25^. NETs are present in multiple diseases, including asthma^26,27^. However, whether EETs contribute to the pathogenesis of asthma because of unique biological activities is not known.

The present study hypothesized that more activated peripheral blood eosinophils contribute to higher levels of EET formation, which leads to enhanced airway inflammation in asthmatic patients, especially SEA. Therefore, the effects of EETs on major inflammatory cells and airway epithelial cells involved in immune responses were evaluated.

## Materials and methods

### Study subjects

The Institutional Review Board of Ajou University Hospital approved this study (AJIRB-GEN-SMP-13-108). All study subjects provided written informed consent at the time of recruitment. We enrolled 20 patients with SEA and 20 patients with non-severe asthma (NSA). All patients were diagnosed based on histories of recurrent episodes of wheezing, dyspnea, cough, sputum production, and evidence of airway hyperresponsiveness to methacholine or reversible airway obstruction that improved with a short-acting β_2_ agonist^28^. Levels of ECP and EDN in serum were measured using the enzyme-linked immunosorbent assays (ELISA) kits (MBL, Sakae, Japan), according to the manufacturer’s instructions. All patients enrolled in this study had a history of asthma exacerbations that required systemic steroids (higher than 15 mg or prednisolone/d for >3 d) and sputum eosinophilia (greater than 3% of sputum leukocytes counted). Patients who were current smokers, exhibited comorbidity with chronic obstructive pulmonary disease or other chronic diseases affecting asthma outcomes were excluded. The SEA group was defined, as described previously^29^.

### Isolation of peripheral blood eosinophils

Venous blood was collected from study subjects in stable, not exacerbation, states. Samples were collected in BD Vacutainer^®^ tubes containing acid citrate dextrose solution (BD Biosciences, Franklin Lakes, NJ, USA), stored at room temperature, and processed within 2 h of collection. Blood was layered on a Lymphoprep™ Solution (Axis-Shield, Oslo, Norway), followed by centrifugation at 879 × *g* at 20 °C for 25 min without braking. Eosinophils were isolated from the fraction containing red blood cells and granulocytes using the Eosinophil Isolation Kit and MACS Column (Miltenyi Biotec Inc., Auburn, CA, USA) according to the manufacturer’s instructions.

### Isolation of eosinophil extracellular traps

Peripheral blood eosinophils (1×10^6^) were primed with 25 ng/mL human recombinant IL-5 (Sigma-Aldrich, St. Louis, MO, USA) for 20 min and stimulated with 0.3 μg/mL LPS (Sigma-Aldrich) for 3 h to induce EETs. DNA concentration was measured using the Quant-iT™ PicoGreen^®^ dsDNA kit (Invitrogen, Paisley, UK). Protein concentrations were evaluated using the QuantiPro BCA Assay Kit (Sigma-Aldrich). Cells were stained with 4′,6-diamidino-2-phenylindole and anti-EPO antibody (Cell Signaling, Minneapolis, MN, USA) and analyzed using a Zeiss LSM710 confocal microscope (Carl Zeiss AG, Oberkochen, Germany) to detect EET-forming eosinophils.

### Treatment with dexamethasone, hydroxychloroquine, or antioxidant

Peripheral blood eosinophils from patients with NSA were maintained in serum-free RPMI-1640 medium (Thermo Fisher Scientific, Waltham, CA, USA) and pretreated with 1 µM dexamethasone (Sigma-Aldrich), 10 µM hydroxychloroquine (Sigma-Aldrich), or 1 µg/mL *N*-Acetyl-l-cysteine (Sigma-Aldrich) for 15 min prior to IL-5 and LPS stimulation. Cells were treated with 10 µM H_2_DCFDA (Thermo Fisher Scientific) and incubated for 30 min at 37 ℃ to detect intracellular reactive oxygen species. Propidium iodide (1 µg/mL; Thermo Fisher Scientific) was added, and signals were read at a wavelength of 620 nm.

### Stimulation of airway epithelial cells

A549 cells, BEAS-2B cells, and human primary small airway epithelial cells (American Type Culture Collection, Manassas, VA, USA) were purchased. A549 cells and BEAS-2B cells were cultured in RPMI-1640 medium supplemented with 10% fetal bovine serum, 100 IU/mL penicillin and 50 µg/mL streptomycin (all components from Thermo Fisher Scientific). Human primary small airway epithelial cells were cultured in airway epithelial cell basal medium supplemented with 2.4 mM L-alanyl-L glutamine, 0.4% extract P, 0.5% plasma protein fraction, 1.0 µM epinephrine, 5 µg/mL transferrin, 10 nM T3, 5 µg/mL hydrocortisone, 5 ng/mL human recombinant epidermal growth factor, and 5 µg/mL human recombinant insulin (all components were provided in a Small Airway Epithelial Cell Growth Kit, ATCC). Cells were treated with 10 nM phorbol myristate acetate (Sigma-Aldrich) or 5 µg/mL EETs for 24 h. IL-8 (Endogen, Woburn, MA, USA) and IL-6 (R&D Systems, Minneapolis, MN, USA) concentrations in culture supernatants were measured using ELISA kits according to the manufacturer’s protocol.

### Evaluation of cell detachment

Cell numbers were assessed using a cell counting kit (CCK)-8 (Dojindo Molecular Technologies, Kumamoto, Japan) according to the manufacturer’s protocol. Culture plates were washed once with PBS to eliminate detached cells prior to addition of the CCK-8 solution and evaluate cell detachment. The %cell detachment = [(number of cells in the unwashed plates−number of cells in the washed plate)/number of cells in unwashed plates] × 100%.

### Epithelial permeability assay

A549 cells were cultured on collagen-coated transwell chambers and treated with EETs for 24 h. The apical chamber was washed with Hank’s balanced salt solution, and 1 mg/mL FITC-dextran (Sigma-Aldrich) was added for 6 h. Media from the basal chamber were collected, and fluorescence intensity was measured (excitation, 490 nm and emission, 530 nm).

### β-hexosaminidase release test

LAD-2 cells (National Institute of Allergy and Infectious Diseases, Bethesda, MD, USA) were maintained in StemPro-34 medium (Life Technologies, Grand Island, NY, USA) supplemented with 2 mM l-glutamine, 100 U/mL penicillin, 50 μg/mL streptomycin, and 100 ng/mL recombinant human stem cell factor (R&D Systems). LAD-2 cells were sensitized using 100 ng/mL biotinylated-IgE (BioPorto Diagnostics, Hellerup, Denmark) with or without 5 µg/mL EETs. Cells were stimulated with 100 ng/mL streptavidin peroxidase (Sigma-Aldrich) in Tyrode’s buffer containing 0.1% BSA. Total β-hexosaminidase was obtained via lysing of LAD-2 cells in 0.1% Triton X-100 in PBS. The supernatants were collected and incubated with an equal volume of 4 mM *p*-nitrophenyl *N*-acetyl-β-d-glucosamide (Sigma-Aldrich) in citrate buffer for 1 h. The reactions were stopped via the addition of 0.4 M glycine buffer, and signals were read at a wavelength of 405 nm. Levels of TNF-α and MCP-1 (R&D Systems) were measured using ELISA kits according to the manufacturer’s protocol.

### Statistical analysis

Student’s *t* test was used to compare continuous variables, and Pearson’s chi-square test was used for categorical variables. Statistical correlations were analyzed using Pearson’s rank coefficient. All statistical analyses were performed using IBM SPSS software version 22.0 (IBM Corp., Armonk, NY, USA). *P* values < 0.05 were considered to indicate statistical significance. GraphPad Prism 5.0 software (GraphPad Inc., San Diego, CA, USA) was used to create graphs.

## Results

### Demographic data from study subjects

Table 1 shows the clinical characteristics of the study subjects. Mean baseline FEV_1_ and PC_20_ methacholine levels were significantly lower in the SEA group than in the NSA group (*P* *=* 0.001, *P* = 0.013). Sputum eosinophils (%) and serum EDN levels were markedly higher in patients with SEA than in those with NSA (*P* *=* 0.024, *P* *=* 0.005). Serum ECP levels tended to be higher in SEA.

**Table 1:** Clinical characteristics of the study subjects

Variables	NSA (*n* = 20)	SEA (*n* = 20)	*P* value
Age (year)	54.9 ± 15.6	57.5 ± 6.5	0.506
Sex (Male, %)	50.0	30.0	0.333
Atopy (%)	60.0	65.0	1.000
Baseline FEV_1_ (%)	100.1 ± 10.9	72.5 ± 15.9	**0.001**
PC_20_, methacholine (mg/mL)	12.9 ± 10.8	2.5 ± 1.4	**0.013**
Serum total IgE (IU/ml)	302.3 ± 334.4	249.3 ± 184.8	0.540
Total eosinophil count (/μL)	380.1 ± 661.8	389.0 ± 399.2	0.959
Sputum eosinophils (%)	16.1 ± 26.9	40.9 ± 38.7	**0.024**
Sputum neutrophils (%)	67.3 ± 30.5	48.2 ± 36.7	0.082
Serum ECP (ng/mL)	74.5 ± 56.5	93.9 ± 68.6	0.398
Serum EDN (ng/mL)	28.5 ± 12.2	44.9 ± 16.4	**0.005**

Values are given as *n* (%) for categorical variables and as the mean ± SD for continuous variables

*P* values were calculated using the Pearson chi-square test for categorical variables and Student’s *t* test for continuous variables

*FEV*_1_ forced exhaled volume at 1 s, *PC*_20_ concentration of methacholine to induce a 20% decline in FEV_1_, *ECP* eosinophil cationic protein, *EDN* eosinophil-derived neurotoxin, *NSA* non-severe asthma, *SEA* severe eosinophilic asthma

Bold values indicate the clinical variables which show statistical significance

### Characterization of EETs from asthmatic patients

**Fig. 1:**
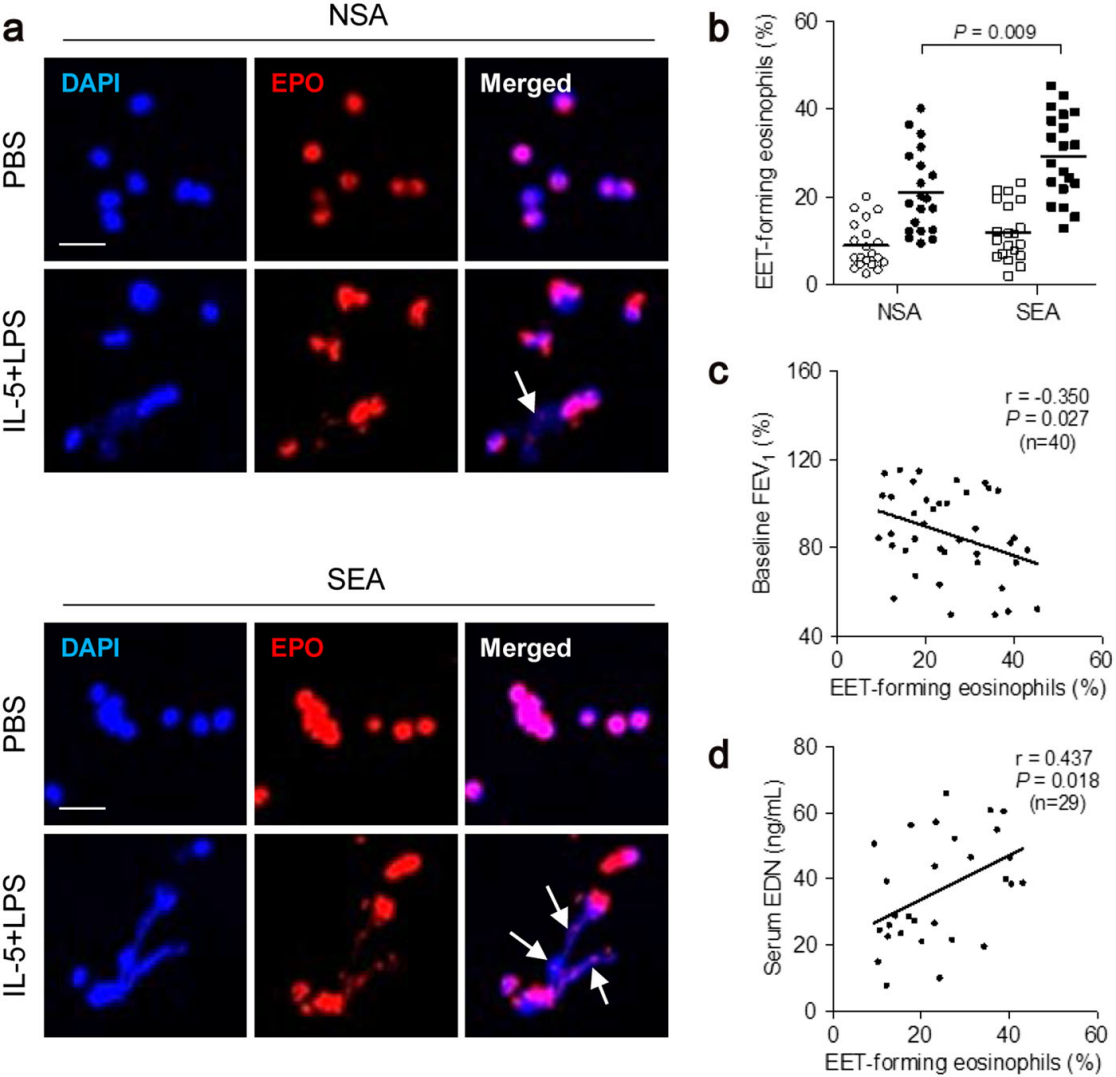
Characteristics of EETs from peripheral blood eosinophils of patients with NSA and SEA. **a** Induction of EET formation (white arrows). Scale bar, 50 µm. **b** Comparison of the percentage of EET-forming eosinophils with (closed) or without (open) IL-5 and LPS stimulation. Associations between EET formation and **c** baseline FEV_1_/**d** serum EDN levels. The data are presented as Pearson correlation coefficient *r* (*P* value)

Peripheral blood eosinophils were treated with IL-5 and LPS to investigate EET formation. The percentage of EET-forming eosinophils was measured using immunostaining (Fig. 1a). Eosinophils were stimulated, and the proportion of cells releasing EETs was significantly higher in the SEA group than in the NSA group. However, unstimulated eosinophils exhibited no significant differences between the 2 groups (Fig. 1b), which indicates that eosinophils from patients with SEA may be more easily activated to produce EETs. The relationship of EET-forming eosinophils with clinical parameters in asthma was investigated. A negative correlation between EET formation and baseline FEV_1_ % predicted values was observed (*r* = –0.350, *P* = 0.027; Fig. 1c), and a positive correlation was found between EET formation and serum EDN levels (*r* = 0.437, *P* *=* 0.018; Fig. 1d).

### Association between EET formation and ROS production

**Fig. 2:**
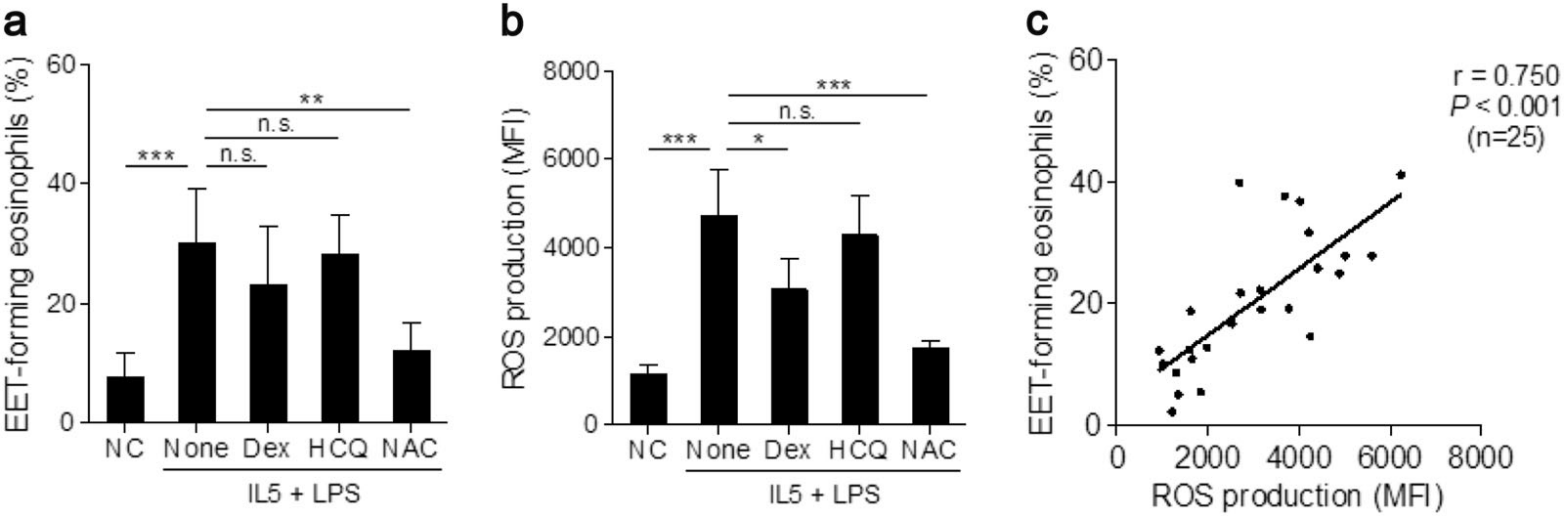
Reactive oxygen species (ROS)-dependent EET production from peripheral blood eosinophils of patients with NSA. Effects of dexamethasone (Dex), hydroxychloroquine (HCQ) or *N*-acetyl-l-cysteine (NAC) on **a** EET formation and **b** ROS production. Data are presented as the mean ± SD, *n* = 5. *P *<* 0.05, **P *<* 0.01 and ***P *<* 0.001 obtained using one-way ANOVA with Bonferroni’s post hoc test. n.s., not significant. **c** A correlation between ROS and EET production. The data are presented as Pearson correlation coefficient *r* (*P* value)

Peripheral blood eosinophils from patients with NSA were treated with various drugs to investigate the regulation of EET formation via inhibition of ROS production. *N*-acetyl-l-cysteine (an antioxidant) markedly reduced the percentage of EET-forming eosinophils stimulated with IL-5 and LPS. Dexamethasone (a representative anti-inflammatory drug used in the treatment of asthma) and hydroxychloroquine (an autophagy inhibitor) produced no significant effect (Fig. 2a). *N*-acetyl-l-cysteine, but not hydroxychloroquine, also attenuated ROS production (Fig. 2b). EET formation was positively correlated with ROS production (*r* = 0.750, *P* *<* 0.001; Fig. 2c), which indicates that regulation of ROS production effectively controls EET-forming eosinophils.

### Autocrine function of EETs in eosinophil activation

**Fig. 3:**
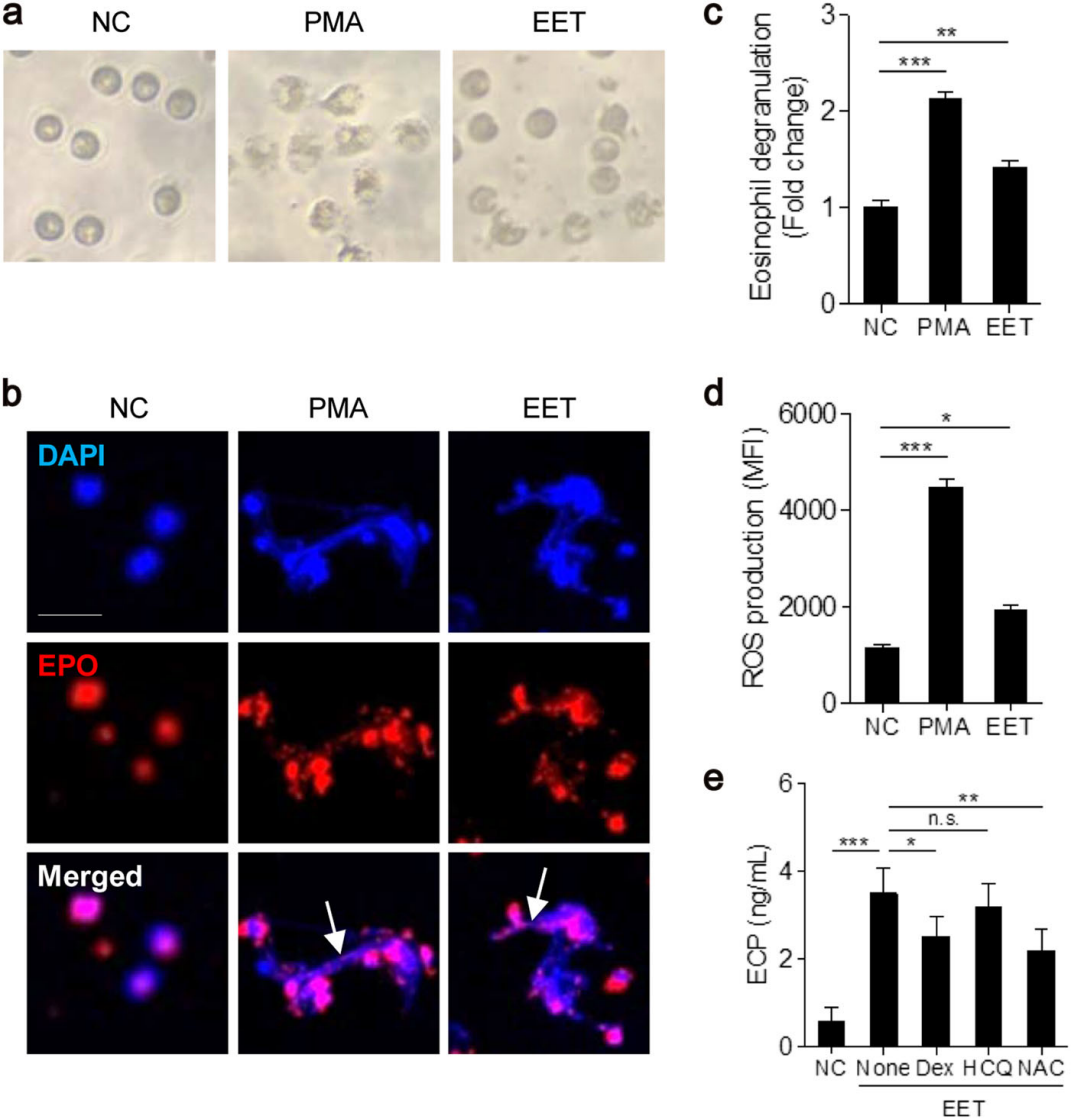
Autocrine function of EETs on eosinophil activation. **a** Morphological changes of peripheral blood eosinophils from patients with NSA after 100 nM phorbol myristate acetate (PMA) or 5 µg/mL EET (from NSA) treatment. **b** Immunofluorescence staining of released EETs (white arrows). Scale bar, 10 µm. **c** Eosinophil degranulation. **d** ROS production. **e** Effect of Dex, HCQ and NAC on eosinophil production of ECP. Data are presented as the mean, *n* = 5. *P *<* 0.05, **P *<* 0.01, and ***P *<* 0.001 obtained using one-way ANOVA with Bonferroni’s post hoc test. n.s., not significant

The current study investigated whether EETs enhanced eosinophilic inflammation via formation of a vicious cycle with autocrine function. Peripheral blood eosinophils were treated with EETs, and the effect of EETs was compared with that of phorbol myristate acetate (PMA), which is a strong stimulator of eosinophils. PMA and EETs produced morphological changes in eosinophils, which exhibited a hole on the cell surface (Fig. 3a). Confocal images revealed that EETs generated EET formation from eosinophils themselves (Fig. 3b). EET treatment also significantly elevated cell degranulation and ROS production. However, the effect was weaker for EET treatment than for PMA treatment (Fig. 3c, d). The present study also evaluated the effects of dexamethasone, hydroxychloroquine or *N*-acetyl-l-cysteine on EET-treated eosinophils. Dexamethasone and *N*-acetyl-l-cysteine attenuated the production of granule proteins (Fig. 3e).

### EET-induced airway epithelial cell detachment and immune responses

**Fig. 4:**
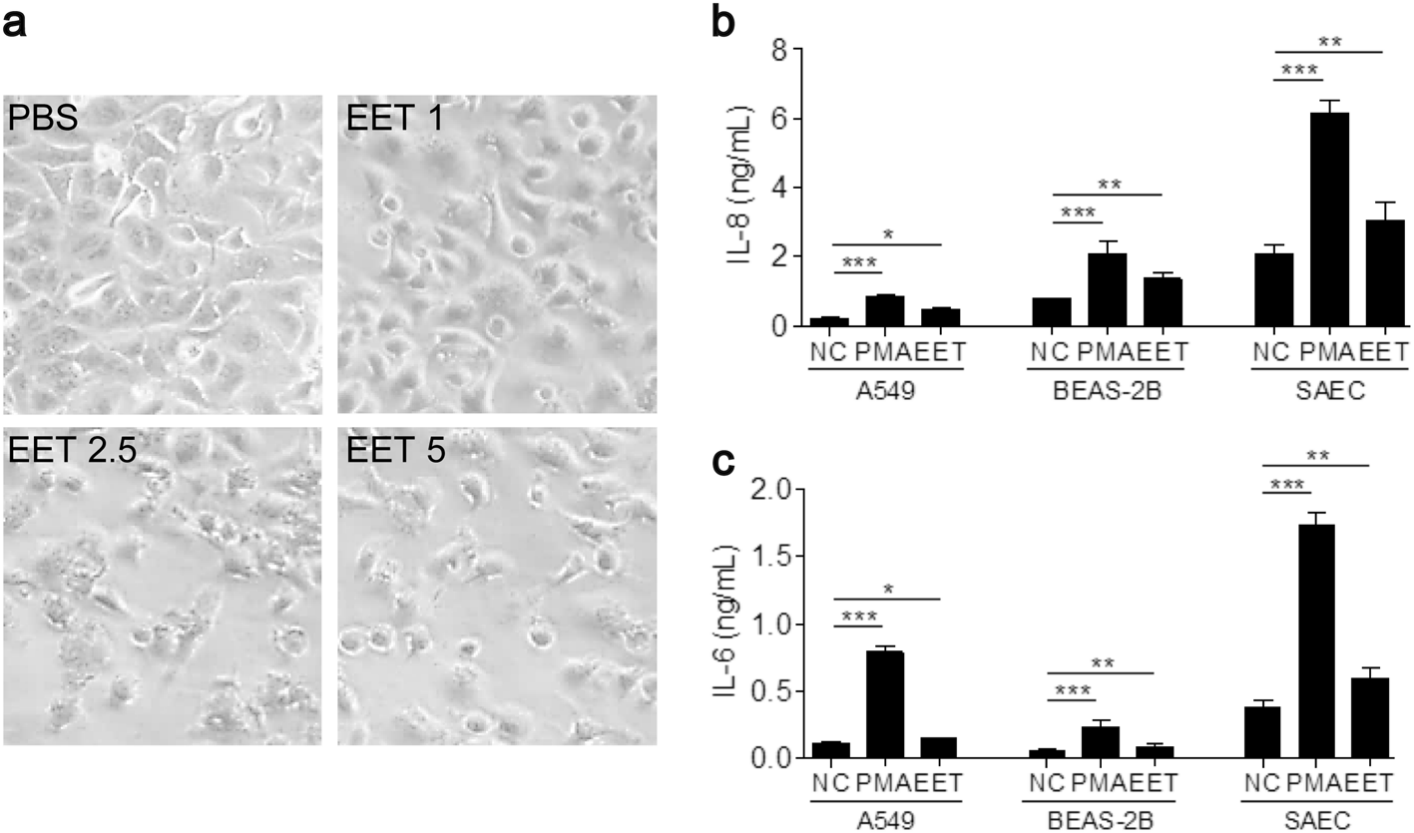
Effect of EETs on airway epithelial cells. **a** Changes in A549 cell morphology following EET (from NSA) treatment in a dose-dependent manner. Concentrations of **b** IL-8 and **c** IL-6 released from A549, BEAS-2B, and human primary small airway epithelial cells (SAEC) treated with 5 µg/mL EETs. Data are presented as the mean ± SD, *n* = 5. *P < 0.05, **P < 0.01, and ***P < 0.001 were obtained using one-way ANOVA with Bonferroni’s post hoc test

EETs altered the morphology and density of A549 cells in a dose-dependent manner (Fig. 4a). EETs induced >10% cell detachment (Supplementary Figure 1a). Airway epithelial cells were cultured in transwells, and the fluorescence intensity of FITC-dextran in the basal chamber was measured to confirm the effect of EETs on epithelial permeability. EET-treated A549 cells exhibited greater fluorescence than untreated cells, which indicates that EETs increased epithelial permeability (Supplementary Figure 1b). The present study evaluated the capacity of EETs to induce pro-inflammatory cytokine production from airway epithelial cells. EETs significantly enhanced IL-8 and IL-6 release from A549, BEAS-2B and human primary small airway epithelial cells (Fig. 4b, c). Dexamethasone, an anti-MBP antibody and anti-ECP antibody were used to determine whether EET-mediated immune responses could be regulated. Dexamethasone and the anti-ECP antibody, but not the anti-MBP antibody, strongly reduced the production of IL-8 from A549 cells (Supplementary Figure 2). These data suggest that cytotoxic granule proteins that are co-localized in EETs are an important factor in inducing airway inflammation from epithelial cells.

### Effect of EETs on mast cell activation

**Fig. 5:**
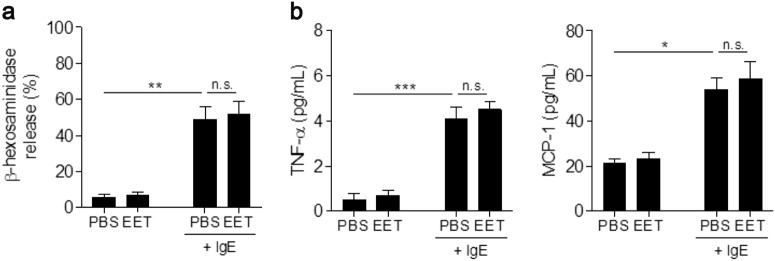
Effect of EETs on mast cell activation. **a** LAD-2 cell degranulation following EET (from NSA) treatment. **b** Levels of TNF-α and MCP-1 released from LAD-2 cells. Data are presented as the mean ± SD, *n* = 3. **P *<* 0.01 was obtained using one-way ANOVA with Bonferroni’s post hoc test. n.s., not significant

LAD-2 cells were treated with EETs in the presence or absence of IgE to evaluate the effect of EETs on mast cell activation. LAD-2 cells stimulated with EETs exhibited no significant differences in cell degranulation compared with controls regardless of the presence of IgE (Fig. 5a), which indicates that EETs do not play a crucial role in the induction of mast cell degranulation. EET treatment did not markedly enhance TNF-α or MCP-1 release from LAD-2 cells (Fig. 5b).

## Discussion

This study is the first report to demonstrate a harmful effect of eosinophils via EET production with an autocrine function and induction of immune responses in asthma. Eosinophils from patients with SEA were more activated than eosinophils from patients with NSA, which increased ROS production and EET formation. In addition, the percentage of EET-forming eosinophils was negatively correlated with baseline FEV_1_. These findings suggest that EETs play a critical role in eosinophilic airway inflammation.

Several previous papers demonstrated the presence of EETs in multiple eosinophilic diseases^15–17^. A previous paper suggested enhanced EET formation in the airway mucosa of asthmatic patients with severe airway inflammation^18^. The present study revealed that EET-forming eosinophils were negatively correlated with baseline FEV_1_ % predicted values and positively correlated with serum EDN levels in asthmatic patients. Peripheral blood eosinophils isolated from patients with SEA exhibited increased EET production following activation with IL-5 and LPS in an ROS-dependent manner. Our results suggest that increased EETs are closely associated with airway inflammation and obstruction in patients with SEA.

Airway epithelial cells are the first-line barrier against antigens in the lungs^30^. These cells are an essential immunomodulator of immune responses to the allergens, viruses and pollutants involved in the pathogenesis of asthma^31^. The present study demonstrated that EETs contributed to the impairment of airway integrity via airway epithelial detachment, which increased epithelial permeability and enhanced airway inflammation in asthmatic airways. Cell death follows cell detachment because airway epithelial cells require adhesion to the basement membrane for survival^32^. We confirmed the pro-inflammatory effect of EETs using IL-8 production from airway epithelial cells. Increased IL-8 concentrations are commonly found in inflammatory sites in patients with respiratory virus infection, chronic obstructive pulmonary diseases or asthma^33^. Many previous studies focused on the inhibition of eosinophil-derived cytotoxic granule proteins, such as MBP and ECP, to regulate immune responses in the airway epithelium^34^. Our data demonstrated that a specific antibody against ECP significantly reduced EET-mediated pro-inflammatory cytokine production from airway epithelial cells. Dexamethasone also significantly decreased IL-8 production, but this result may be a simple anti-inflammatory effect on these cells. These findings suggest that further investigational drugs are needed to suppress the EET-epithelial cell axis in patients with SEA.

Mast cells contribute to the pathogenesis of SEA. Mast cells produce histamine and type 2 cytokines, such as IL-4, IL-5, and IL-13, which regulate the development and activation of eosinophilic inflammation^35^. Mast cells play a crucial role in asthma-related airway inflammation, and the present study investigated the effect of EETs on mast cells. However, EETs did not significantly induce degranulation or cytokine production; hence, IgE may be more important for mast cell stimulation.

The current study has some limitations. First, the pathophysiological function of EETs was not confirmed in vivo. A previous study revealed elevated EET formation in ovalbumin-challenged mice, but the effect of EETs in eosinophilic asthma is not clear^36^. Second, EET-forming eosinophils in the lungs of asthmatic patients were not investigated, which would provide direct evidence for EETs in the pathogenesis of asthma. Further investigations of the detailed underlying molecular mechanisms and new potential therapeutic targets are warranted.

In conclusion, EETs may play an important role in the pathogenesis of eosinophilic airway inflammation via activation of eosinophils and epithelial cells. Therefore, the regulation of EET formation or function may be a novel approach for the treatment of asthmatic patients.

## Electronic supplementary material

Supplementary Information
