# Supplementary material for: Biological function of eosinophil extracellular traps in patients with severe eosinophilic asthma
Source: Exp Mol Med. 2018 Aug 16;50(8):104. doi: 10.1038/s12276-018-0136-8 (PMC6095846; doi:10.1038/s12276-018-0136-8)
Supplement: Supplementary file 1 — Supplementary Information [file 12276_2018_136_MOESM1_ESM.docx]

**Supplementary Information**

**Biological function of eosinophil extracellular traps in patients with severe eosinophilic asthma**

Youngwoo Choi,^1^ Duy Le Pham,^1,2^ Dong-Hyun Lee,^1^ So-Hee Lee,^1^ Seung-Hyun Kim,^3^ and Hae-Sim Park^1,3^

^1^Department of Allergy and Clinical Immunology, Ajou University School of Medicine, Suwon, South Korea

^2^Faculty of Medicine, University of Medicine and Pharmacy, Ho Chi Minh city, Viet Nam

^3^Clinical Trial Center, Ajou University Medical Center, Suwon, South Korea

*These authors contributed equally to this work.

**Corresponding author:** Professor Hae-Sim Park, MD, PhD

Department of Allergy and Clinical Immunology, Ajou University School of Medicine, 164 World cup-ro, Yeongtong-gu, Suwon, Korea.

Tel: +82-31-219-5196,

Fax: +82-31-219-5154,

Email: hspark@ajou.ac.kr


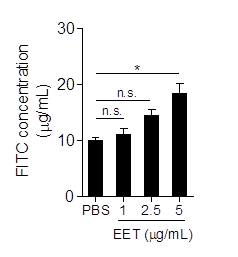

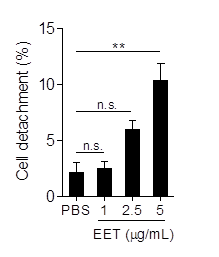


a

b

**Supplementary Figure 1.** Effect of EETs on A549 cell permeability. (a) Cell detachment. (b) FITC-dextran permeability assay. Data are presented as means ± SD, n = 5. **P* < 0.05 and ***P* < 0.01 were obtained by using one-way ANOVA with Bonferroni’s *post hoc* test. n.s., not significant.

**
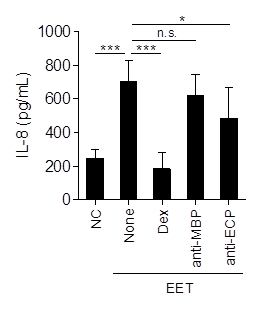
**

**Supplementary Figure 2.** Effect of Dex (1 µM), anti-MBP antibody or anti-ECP antibody (200 µg/mL) on A549 cell treated with EETs (5 µg/mL). Data are presented as means ± SD, n = 5. **P* < 0.05 and ****P* < 0.001 were obtained by using one-way ANOVA with Bonferroni’s *post hoc* test. n.s., not significant.
